# Retraction of: Material and regenerative properties of an osteon-mimetic cortical bone-like scaffold

**DOI:** 10.1093/rb/rbag067

**Published:** 2026-04-29

This is a retraction of: Danial Barati, Ozan Karaman, Seyedsina Moeinzadeh, Safaa Kader, Esmaiel Jabbari, Material and regenerative properties of an osteon-mimetic cortical bone-like scaffold, *Regenerative Biomaterials*, Volume 6, Issue 2, March 2019, Pages 89–98, https://doi.org/10.1093/rb/rbz008.

In January 2026, a reader alerted the Editors to similarities between a number of the images presented in the article and those published by the same author group in *Langmuir* in 2015 and *Biomacromolecules* in 2019. These concerns were also raised via PubPeer (https://pubpeer.com/publications/0B53F38D11491D25C54E99A4500235).

Specifically, in Figure 2A, the SEM Image was duplicated from Figure 4B in Danial Barati, Joshua D. Walters, Seyed Ramin, Pajoum Shariati, Seyedsina Moeinzadeh, Esmaiel Jabbari, Effect of Organic Acids on Calcium Phosphate Nucleation and Osteogenic Differentiation of Human Mesenchymal Stem Cells on Peptide Functionalized Nanofibers, *Langmuir*, Volume 31, Issue 18, May 2015, Pages 5130–5140, 10.1021/acs.langmuir.5b00615. Figure 2A shares features with panels in Figure 4B representing different materials, despite scale bars that indicated different sample properties. In Figure 7B, the CD31 and β-actin blot images were reused from Figure 12G in Safaa Kader, Mehri Monavarian, Danial Barati, Seyedsina Moeinzadeh, Thomas M. Makris, Esmaiel Jabbari, Plasmin-Cleavable Nanoparticles for On-Demand Release of Morphogens in Vascularized Osteogenesis, *Biomacromolecules*, Volume 20, Issue 8, August 2019, Pages 2973–2988, 10.1021/acs.biomac.9b00532.

The Editors contacted the corresponding author who explained that the paper had been published seven years ago and they were unable to retrieve the raw images of Figure 2A and Figure 7D.

The Editors investigated the concerns raised and have decided to retract this paper because the image duplication described above, and the authors’ inability to supply the raw images, undermine their confidence in the results and conclusions reported in the paper. The journal notified all authors of this decision prior to publication of this retraction notice. This decision has been made in accordance with guidance developed by the Committee on Publication Ethics (COPE).

